# The genetics and development of mandibles and hypopharyngeal sclerite and cornua in larvae of *Drosophila gaucha*

**DOI:** 10.1371/journal.pone.0185054

**Published:** 2017-10-18

**Authors:** Eduardo Alvarez, Francisco Del Pino, Lilian Jara, Raúl Godoy-Herrera

**Affiliations:** Programa de Genética Humana, ICBM, Facultad de Medicina, Universidad de Chile, Santiago, Chile; Natural Resources Canada, CANADA

## Abstract

The genetics and epigenetic processes associated with morphological organization are a principal aim of biology, ranging from cohesion between cells to shape and size of organisms. We investigate the post-embryonic development of Hypopharyngeal sclerite and cornua HPC and mandibles M of *Drosophila gaucha* larva. Integrated functioning of these Cephalopharyngeal skeleton parts of *D*. *gaucha* larva is essential for food acquisition, participating in locomotion and microhabitat selection. We examined two isolates by recording the growth of the HPC and M every 24 h for 8 days in parental, F_**1**_, F_**2**_ and backcross larvae. In F_**1**_ larvae, the HPC and M growth was similar to the parental. In F_**2**_ and backcross larvae, the growth was slower. Epistasis and dominance are the principal sources upon which the growth of HPC and M are based. Pleiotropic genes seem also to be involved in integrating the development of M and HPC. Our data suggest that hybridization of the isolates modified epigenetic processes involved in the development of those morphological structures of *D*. *gaucha* larva.

## Introduction

Animals have highly sophisticated molecular mechanisms to regulate the development of cell, organ and body size [1, 2]. Animal cells synthesize a variety of chemical signals to communicate to form collaborative tissues and organs [3]. Such linkage ensures that morphological organization is expressed in coordinate manner in relation to environment [2, 4, 5]. Therefore, it is important to investigate growth and integration among body parts to understand the shape and size of organisms [6]. Of particular interest is the post-embryonic development of body parts that act coordinately and participating in ecological functions intimately associated with feeding rates and selection of microhabitats, and ultimately, with biological fitness [7, 8]. As development proceeds, epigenetic relationships between genes may be modified, and similarly, the ecological demands of an individual change during its life history. Consequently, it is important link epigenetic processes with ecology and evolution of populations by investigating isolates living in heterogeneous and changing environments. Inter-population differences in the development of bodily parts may indicate epigenetic differentiation in gene expression, suggesting that gene regulation is under natural selection action [9]. Additionally, population investigations of epigenetic processes aid to understand phenotypic plasticity, and adaptation and colonization of new habitats [10,11]. In this study, we investigate the proposition that the post-embryonic development of Mandibles M and Hypopharyngeal sclerite and Cornua HPC of *Drosophila* larva is associated with the ecology of breeding sites. This notion suggests that those parts of cephalopharyngeal skeleton of *Drosophila* larva [12] act coordinately to shovel food into the gut of larvae. The structures also participate in digging and tunneling into substratum serving as a fulcrum to facilitate larval body movements [13–15].

The changing features of variable *Drosophila* breeding sites [16] may interfere with the development of HPC and M, obstructing the coordination of these body parts. We conjectured that the progress of these morphological parts of *Drosophila* larva should be genetically canalized to originate standard shapes that facilitate interactions among them. We hypothesized that epigenetic interactions expressed in epistasis and pleiotropic phenomena could be key in the development and integration of HPC and M of *Drosophila* larva. Here, we focus on the genetics and development of those parts of Cephalopharyngeal skeleton by examining two natural populations of *Drosophila gaucha* and discussing the role of epigenetic mechanisms in such development.

Elevator and depressor muscle tendons attach via apodemes to the M base and HPC. A hinge joint also links these bodily structures allowing the M to move along on an inclined or horizontal plane [12]. The apical tooth of M in *Drosophila* larva is curve, long and wide, and small teeth are usually present [17], originating a structure fitted for stabbing the substratum [18]. Thus, integrated function of HPC and M combined with muscle contractions of the body walls ensuring that *Drosophila* larva navigate through the substrate searching for food to complete its development, favoring the adaptation of these individuals to environments that change their features in a short time [16].

*D*. *gaucha* larvae breed on decaying cladodes of prickly pear, *Opuntia ficus-indicus* [19]. Because the plant has an extended geographical distribution, *D*. *gaucha* has established populations in a variety of environments [20]. The larval period of *D*. *gaucha* is twice that of *Drosophila melanogaster* at the same temperature [21]. Therefore, *D*. *gaucha* larva is a good model to substantiate the effect of environmental, genotypic and epigenetic changes on the post-embryonic development of poly-functional body parts that act in a coordinated fashion.

## Results

### The growth in length of mandibles

**Fig 1:**
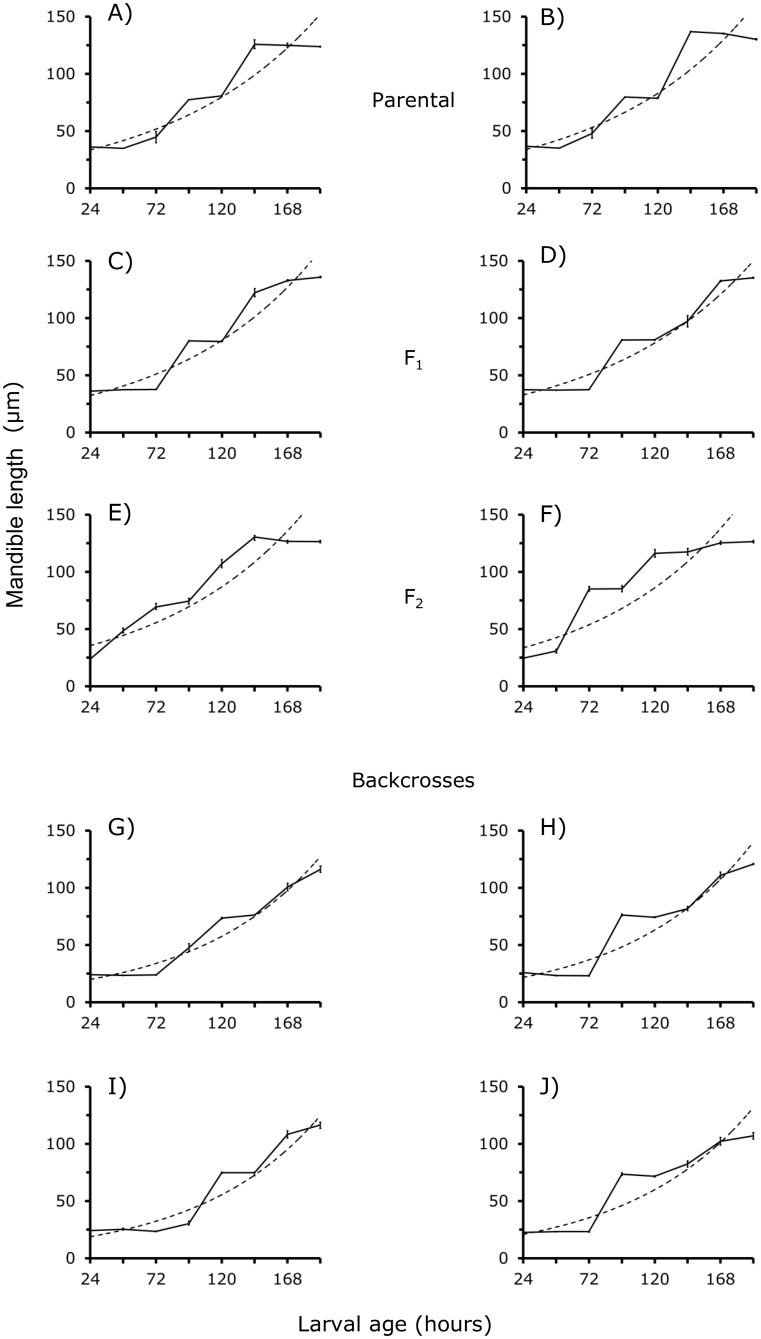
A—J. Growth in length (μm) of mandibles M in *D*. *gaucha* larvae (solid line). The exponential function describing the growth of this body part is shown by the broken line. BA parental strain, A. CJ parental strain, B. Reciprocal F_**1,**_ C and D. Reciprocal F_**2**_, E and F. Reciprocal backcrosses, G—J. For further details see Materials and methods and Tables 2 and 3. When standard errors are not shown is because they are too small.

Fig 1A–1J shows the growth in length (μm) of larval mandibles M of the BA and CJ parental strains and the reciprocal F_**1**_'s, F_**2**_'s and backcrosses between 24 to 192 h of larval age. As the larval development elapses, the length of the M increases stepwise according to an exponential function in larvae of the parental and reciprocal F_**1**_ generations (Fig 1A–1D). By contrast, in F_**2**_ and backcross larvae, the growth in length of M tends to be exponential, but a decrease in size of steps is noted (Fig 1E–1J and Table 1). These findings suggest that recombination of BA and CJ genotypes in the F_**2**_ and backcross generations modified the development pathway of the M (Fig 1E–1J and Table 1).

**Table 1:** Exponential equations computed to the growth in length and width of mandibles and HPC in larvae of the BA and CJ parental strains, reciprocal F_1_, reciprocal F_2_ and backcrosses, *D*. *gaucha*. The larvae were 24, 48, 72, 96, 120, 144, 168, and 192 h of age (N = 50 individuals per group of genotypes and larval age; see Materials and methods). For all crosses, the first parent shown is the female.

Genotype group	Equations		
	mandible		HPC
	length	width	Length
Parental			
BA strain	27.14e^0.009**x**^	5.08e^0.1**x**^	136.27e^0.09**x**^
CJ strain	27.18e^0.009**x**^	5.42e^0.1**x**^	144.76e^0.09**x**^
Reciprocal F_1_			
BA x CJ	25.81e^0.009**x**^	4.93e^0.1**x**^	146.70e^0.09**x**^
CJ x BA	26.48e^0.009**x**^	4.86e^0.1**x**^	146.55e^0.09x^
Reciprocal F_2_			
(BA x CJ) x (BA x CJ)	2 8.48e^0.0009**x**^	9.08e^0.008**x**^	163.19e^0.007**x**^
(CJ x BA) x (CJ x BA)	26.60e^0.0001**x**^	8.92e^0.008**x**^	156.29e^0.007**x**^
Reciprocal backcrosses			
(BA x CJ) x BA	15.34e^0.0001**x**^	4.56e^0.001**x**^	115.59e^0.007**x**^
BA x (BA x CJ)	16.70e^0.0001**x**^	4.89e^0.001**x**^	114.93e^0.007**x**^
(CJ x BA) x CJ	14.44e^0.00012**x**^	4.48e^0.009**x**^	108.90e^0.007**x**^
CJ x (CJ x BA)	16.12e^0.00011**x**^	4.40e^0.009**x**^	117.96e^0.006**x**^

Exponential function describes satisfactorily the growth in length of the M in the parental, F_**1**_, F_**2**_ and backcross generations (*R*^*2*^ coefficients of determination in S3 Table). The calculated equations are presented in Table 1. One-way ANOVA for differences between the BA and CJ parental slopes yielded a *F*_**1, 98**_-value = 0.51, *P* > 0.05, *NS*. The same analysis for slopes of the reciprocal F_**1**_, F_2_ and the four types of backcrosses yielded no significant differences: (i) between the F_**1**_'s, *F*_***1*, *98***_-value = 0.52, *P* > 0.05, *NS*, (ii) between the F_**2**_'s, *F*_***1*, *98***_-value = 1.26, *P* > 0.05, *NS*, (iii) between the four backcrosses, *F*_***3*, *196***_-value = 2.39, *P* > 0.05, *NS*. We infer that differences in the X chromosome and cytoplasm have a negligible role in the length-wise growth of M in *D*. *gaucha* larvae.

Moreover, the slopes of the segregating generations tend to be smaller than those of the parental and F_**1**_ generations (Table 1). For example, in the BA and CJ parental strains, the slopes are 27.14e^**0.009X**^ and 27.18e^**0.009X**^, respectively. In the F_2_ obtained to cross (BA mother x CJ father) mother and (BA mother x CJ father) father, the value is 28.48e^**0.0009X**^ (Table 1). The comparison of parental and F_**1**_’s slopes yielded a *F*_**3, 196**_-value = 0.22, *P* > 0.05, *NS*. The parental and the two F_**2**_'s produced a *F*_***3*, *196***_-value = 10.09, *P* < 0.01, and the parental against the four backcrosses provided a *F*_***5*, *294***_ = 32.15, *P* < 0.001. The analysis confirmed that the introgression of BA and CJ gene pools introduced substantial changes in the development of the M of larvae of the recombinant generations.

### The growth in width of mandible

The growth in width of mandible was previously published as Figure 2 in [22]. It is shown in S1 Fig. Here we comment some principal points of that Fig, and we provide statistical and genetic analysis. The growth in width (μm) of M is exponential and stepwise in BA and CJ and F_**1**_ larvae of *D*. *gaucha* (S1A–S1D Fig). This pattern tends to change to lose some steps in larvae of the F_**2**_ and backcross generations (S1E–S1J Fig). These findings support our contention that the introgression of BA and CJ genes altered the growth of M in F_**2**_ and backcross larvae.

The exponential function effectively describes the width-wise growth of *D*. *gaucha* larvae M (*R*^*2*^ = 86.56%). The equations listed in Table 1 suggest that the width-wise growth of M of F_**2**_ and backcross larvae is slower than that of parental and F_**1**_ larvae (ANOVA were: (i) between BA and CJ parental strains, *F*_***1*, *98***_-value = 0.65, *P* > 0.05, *NS*; (ii) between the parental strains and F_**1**_´s, *F*_***3*,*196***_-value = 0.82, *P* > 0.05, *NS*; (iii) between the parental strains and F_**2**_'s, *F*_***3*,*196***_-value = 10.39, *P* < 0.01; (iv) between the parental strains and backcrosses, *F*_***5*, *294***_-value = 12.36, *P* < 0.05). We conclude that in the F_**2**_ and backcross generations, genetic introgression of the BA and CJ strains tends to delay M growth.

### The growth in length of HPC

**Fig 2:**
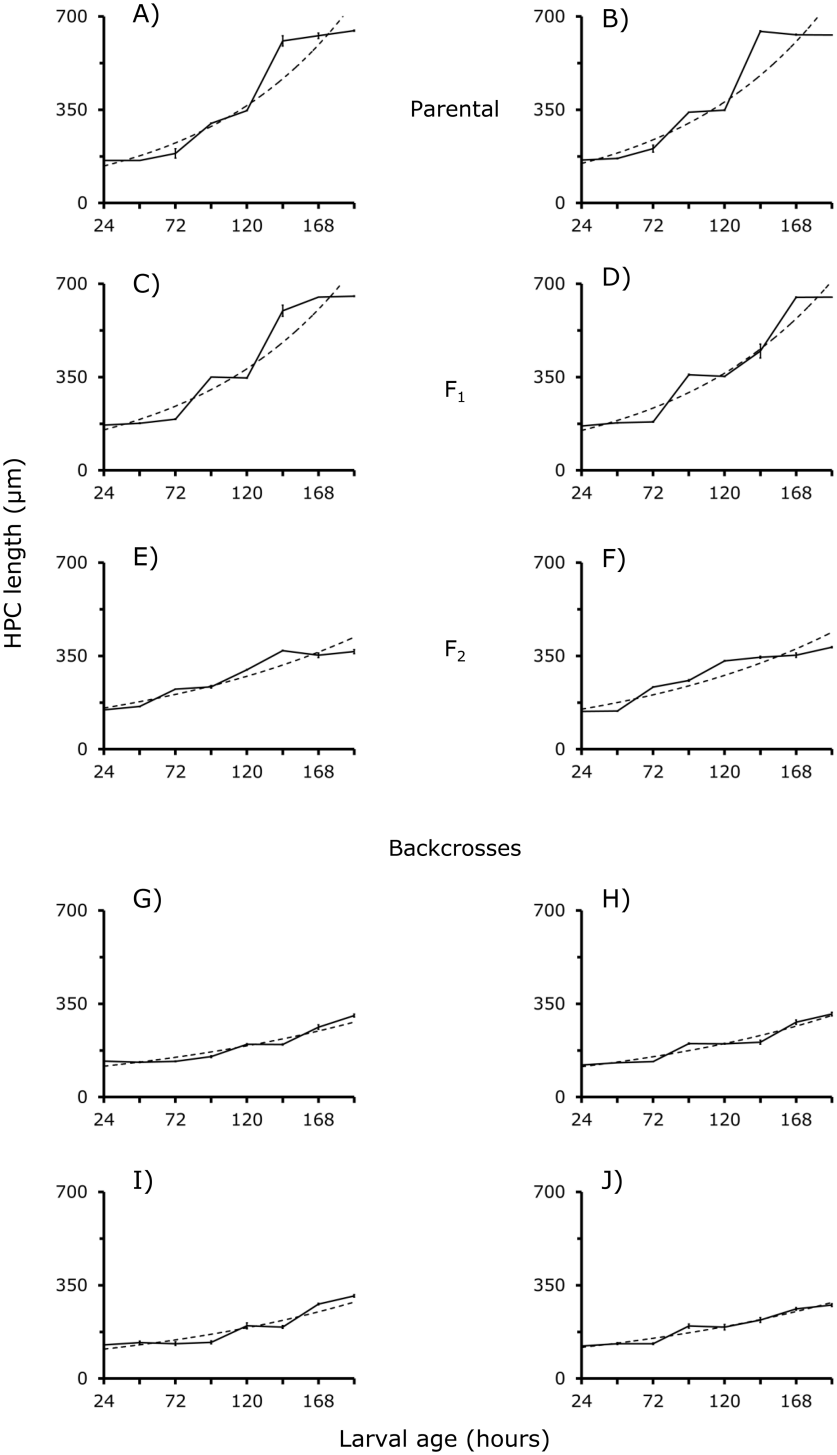
A–J. Growth in length (μm) of HPC in BA, A; CJ, B; F_1_, C and D; F_2_, E and F, and backcross larvae, G–J, of *D*. *gaucha*. For further details see Fig 4.

The growth in length (μm) of HPC is stepwise in BA and CJ and F_**1**_ larvae (Fig 2A–2D). These abrupt jumps in size tend to decrease in the F_**2**_ and backcross larvae (Fig 2E–2J). Taken together, these results with those shown in Fig 1 suggest that hybridization among the BA and CJ strains introduces deep changes in the development of those two parts of head of *D*. *gaucha* larvae. The exponential function effectively describes the HPC growth length (*R*^***2***^ = 92.71). Moreover, the growth slopes of the BA and CJ parental strains and F_**1**_ larval HPC are statistically similar (*F*_***3*,*196***_-value = 0.68, *P* > 0.05, *NS*), but different than those of the F_**2**_ (*F*_*3*, *196*_-value = 45.78, *P* > 0.01) and backcross larvae (*F*_***5*, *294***_- value = 75.03, *P* < 0.01). The findings again suggest that recombination of BA and CJ gene pools substantially modified the development of the HPC in *D*. *gaucha* larvae.

### Teeth number

**Fig 3:**
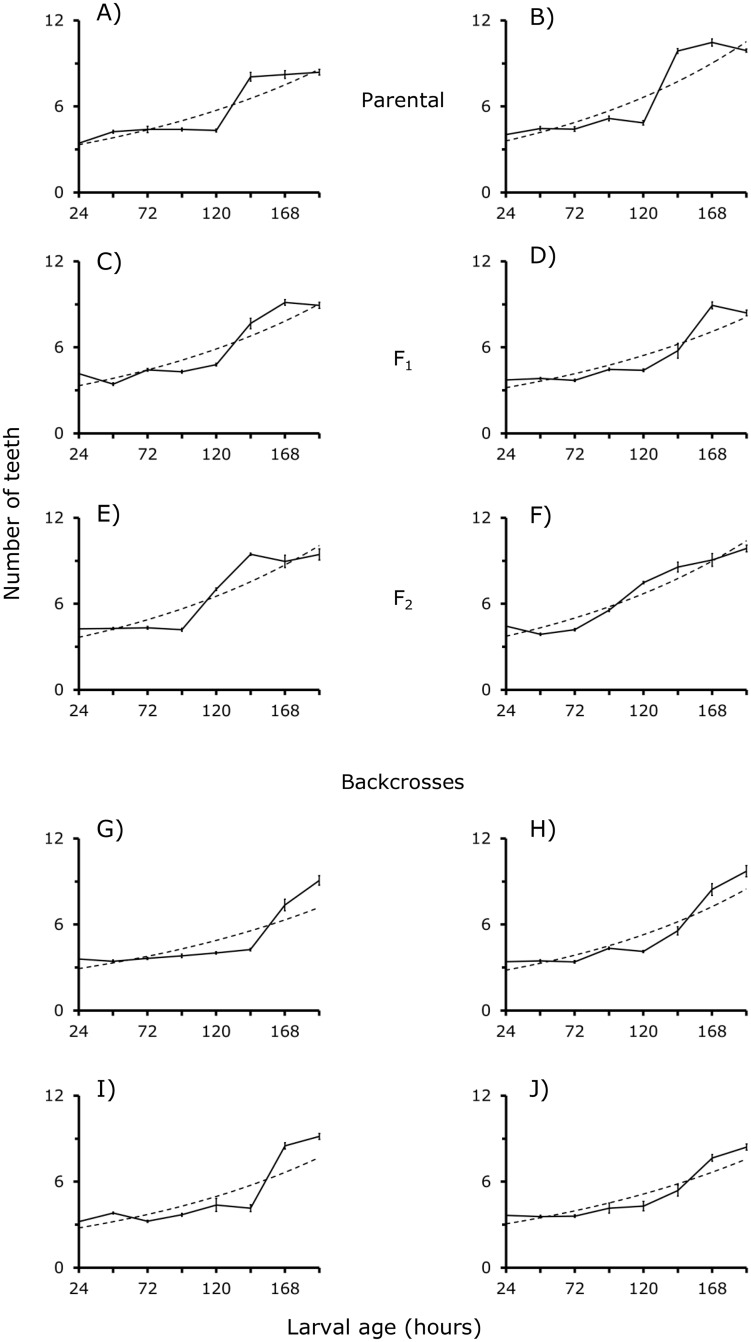
A–J. Number of mandible teeth in BA (A), CJ (B), F_1_ (C and D), F_2_ (E and F) and backcross larvae (G to J) of *D*. *gaucha*. For further details see Fig 4 and Tables 2 and 3.

Fig 3 presents the number of M teeth in larvae of the BA and CJ parental strains and F_**1**_, F_**2**_'s and backcrosses between 24 and 192 h of larval development. Number of teeth in BA, CJ and F_**1**_ larvae is 4 to 5 per M between 24 to 120 h of development (Fig 3A–3D). Between 144 and 192 h of larval development we counted 8 to 10 teeth per M (Fig 3A–3D). By contrast, between 24 and 96 h of development F_**2**_´s larvae exhibited 4 to 5 teeth per M; the number tends to increase progressively until 9 to 10 teeth after 120 h of larval age (Fig 3E–3F). Thus, recombination of BA and CJ gene pools causes a premature emergence of teeth in F_**2**_ larvae (Fig 3E–3F). Interestingly, this situation changed dramatically in the backcross larvae because 4 to 5 teeth were counted in the M of larvae at 24 to 144 h-old- larvae, and 8 to 9 teeth in M of larvae at 168 to 192 h (Fig 3G–3J). Although recombination of BA and CJ genes causes a premature increase of teeth number in M of F_**2**_ larvae, a remarkable delay in emergency of these structures in M of backcross larvae is observed (Fig 3G–3J).

### Genetic analysis

#### Scaling tests

We applied scaling tests (A, B, C), as suggested by Mather and Jinks [23], at 192 h of larval development to analyze genetically phenotypic differences in shape of M and HPC of *D*. *gaucha* larvae obtained from crossing the BA and CJ strains. For M the following values were obtained: A = -22.24 ± 16.35, *t* = 5.50, *df* = 48, *P* < 0.01; B = -44.66 ± 28.32, *t* = 8.40, *df* = 48, *P* < 0.01; C = -21.58 ± 16.18, *t* = 5.36, *df* = 48, *P* < 0,01. Similar results were obtained for HPC: A = 36.65 ± 21.61, *t* = 8.02, *df* = 48, *P*< 0.01; B = 38.52 ± 26.05, *t* = 8.32, *P* < 0.01; C = 19.34 ± 13.23, *t* = 12.03, *P* < 0.01. These values are not compatible with a simple additive-dominance model [23]. In addition, the **χ**^**2**^ test measuring goodness of fit to the additive-dominance model (the joint scale test) also produced the same results as the *t* test (M, **χ**^**2**^ **= 29.29**, *df* = 3, *P* < 0.001; HPC, **χ**^**2**^ **= 26.54**, *df* = 3, *P* < 0.001). Thus, the analysis suggests that epistasis is a principal source in the control of the development of M and HPC of *D*. *gaucha* larva.

#### Estimation of genetic parameters

Table 2 presents estimations of additive, dominance and epistatic parameters for M length and width, HPC length and number of teeth. S2 Table shows *t*-values and statistical significance of the estimations listed in Table 3. Dominance and epistatic genetic interactions are the principal forces that appear to act through the development of these body parts in *D*. *gaucha* larvae (Table 2). Epistasis occurs principally among additive and dominant genes. Dominant and epistatic genes are also associated with number of teeth (Table 2). These estimates are in agreement with data presented in Figs 1–3 and the scaling tests. We infer that the genetic architecture of the shape and size of the HPC and M of *D*. *gaucha* larva reminds to us that exhibited by traits associated with biological fitness as fertility and viability [24].

**Table 2:** Estimation of additive, dominance and epistasis parameters for mandible length and width, CPS length and teeth number in *D*. *gaucha* larvae. The individuals were 192 h of development. Goodness of fit of an additive- dominant-epistatic model to the collected data was performed (Chi-squared test values are listed in S1 Table). Probabilities showing significance of the calculated parameters are given at bottom of the Table, and the corresponding t-values are listed in S2 Table. For all crosses the parental lines were the Buenos Aires (BA) and Campos de Jordan (CJ) strains.

Trait	Parameter					
	[*m*]	[*a*]	[*d*]	[*aa*]	[*ad*]	[*dd*]
Mandible						
Length	167.51 ± 7.44**	4.36 ± 0.32**	132.15 ± 20.61**	39.36 ± 7.44**	—	100.18 ± 13.36**
Width	59.18 ± 3.16**	0.56 ± 0.19**	76.98 ± 7.94**	23.29 ± 3.15**	—	55.18 ± 4.89**
CPS						
Length	930.82 ± 25.19**	8.09 ± 0.93**	1946.69 ± 63.01**	292.07 ± 25.17**	—	1667.56 ± 39.67**
Teeth number	10.56 ± 0.44**	0.76 ± 0.11	1.92 ± 0.57*	1.42 ± 0.46*	2.66 ± 0.98*	—

[m] = Common effects to every genotype; [a] = Additive component; [d] = Dominant effects of means; [aa] = additive x additive interaction; [ad] = additive x dominance interaction; [dd] = dominance x dominance interaction.

*P < 0.01

**P< 0.001

**Table 3:** Mandible M and Hypopharyngeal sclerite and Cornua HPC measurements performed in larvae of *D*. *gaucha*. The larvae were of the CJ and BA strains and F_1_, F_2_ and reciprocal backcrosses. Larvae measured were 24, 48, 72, 96, 120, 144, 168 and 192 h of development (see also Fig 1). Measurements were expressed in micrometers (μm).

Trait measured	Description of measurement
Mandible length	From the apex of the apical tooth to the end of the medial posterior region of the mandible
Mandible width	From the ventral apodeme to the dorsal apodeme
Length of hypopharyngeal sclerite and cornua HPC	From ectostomal sclerite to the ventral arm
Teeth of mandibles	Number of teeth of mandible were counts under microscope in larvae of 24 to 196 h of development

## Discussion

The development of M and HPC and the teeth emergency of Buenos Aires BA, Campos de Jordan CJ, and F_**1**_ larvae of *D*. *gaucha* is very similar (Figs 1A–1D to 3A–3D, see also S1 Fig). The results suggest conservation of the development pathways in the two isolates. In addition, genes of the BA and CJ pools are expressed without any interference in F_**1**_ larvae. By contrast, the results from F_**2**_ and backcrosses suggest substantial changes in gene action (Figs 1F–1J to 3F–3J, see also S1 Fig). In the F_**2**_ and backcross generations, alleles belonging to various loci of one of the populations appear to interact negatively with genes of the other population of *D*. *gaucha* delaying the development of M and HPC. On the other hand, genetic analysis suggested that dominance and epistasis were principal forces controlling the development of M and HPC (Table 3). These findings are all symptoms of coadaptation of the BA and CJ genetic pools [25], suggesting epigenetic canalization for the growth of M, HPC and the emergence of teeth.

To better understand why inter-population recombination caused changes in the growth of M and HPC of F_**2**_ and backcross larvae, but not in F_**1**_ larvae, we focused our attention on *cis*-regulatory sequences of BA and CJ genes. Current understanding of the molecular structure of eukaryotic genes indicates that gene transcription depends on promoters, transcription initiation sites, enhancers, introns and exons. Thus, gene regulatory proteins bind to DNA regulatory sequences, whereas transcription factors assemble on the promoters [3].

These functionally different nucleotides sequences are near to introns and exons, and recombination may occur between regulatory sequences of homologous chromosomes [3, 25, 26]. We conjectured that BA and CJ isolates differ in nucleotide sequences of promoters and *cis*-regulatory DNA sequences at genes that contribute to the development of the M and HPC. We hypothesize that dissimilar gene control regions increase the efficiency of transcription in each population. In female meiosis of the F_**1**_, crossing-over might change some nucleotides regulatory sequences, altering gene transcription patterns, and the development pathways of M and HPC of F_**2**_ and backcross larvae (Figs 1A–1J and 2A–2J). Notably, recombination of the BA and CJ populations resulted in premature emergency of teeth in the F_**2**_ larvae, but there was a delay in the emergence of these structures in backcross larvae (Fig 3A–3J). Future studies should compare nucleotide sequences of *cis*-regulatory elements in natural populations of *D*. *gaucha*.

On the other hand, it is known that distinct chromatin states stimulate or repress gene activity [11]. In the process long non-coding RNAs lncRNAs are key [9, 11]. We conjectured that *cis*-acting lncRNA could control the expression of protein-coding genes located adjacent to their transcription sites; lncRNA-coding loci are often entwined with protein-coding genes [27]. The BA and CJ populations of *D*. *gaucha* could differ in nucleotide sequence and transcript abundance of lncRNA. In the F_2_ and backcross generations changes in the primary sequence of some lncRNA-coding loci could occur. As a result, new interactions could arise between the novel lncRNAs and protein-coding genes affecting the development of M and HPC and teeth emergency (Figs 1E–1J to 3E–3J). Thus, our statistical estimations of additive, dominance and epistatic genetic variance of M, HPC and teeth number based on performance covariances between relatives may reflect changes in gene regulatory networks.

**Fig 4:**
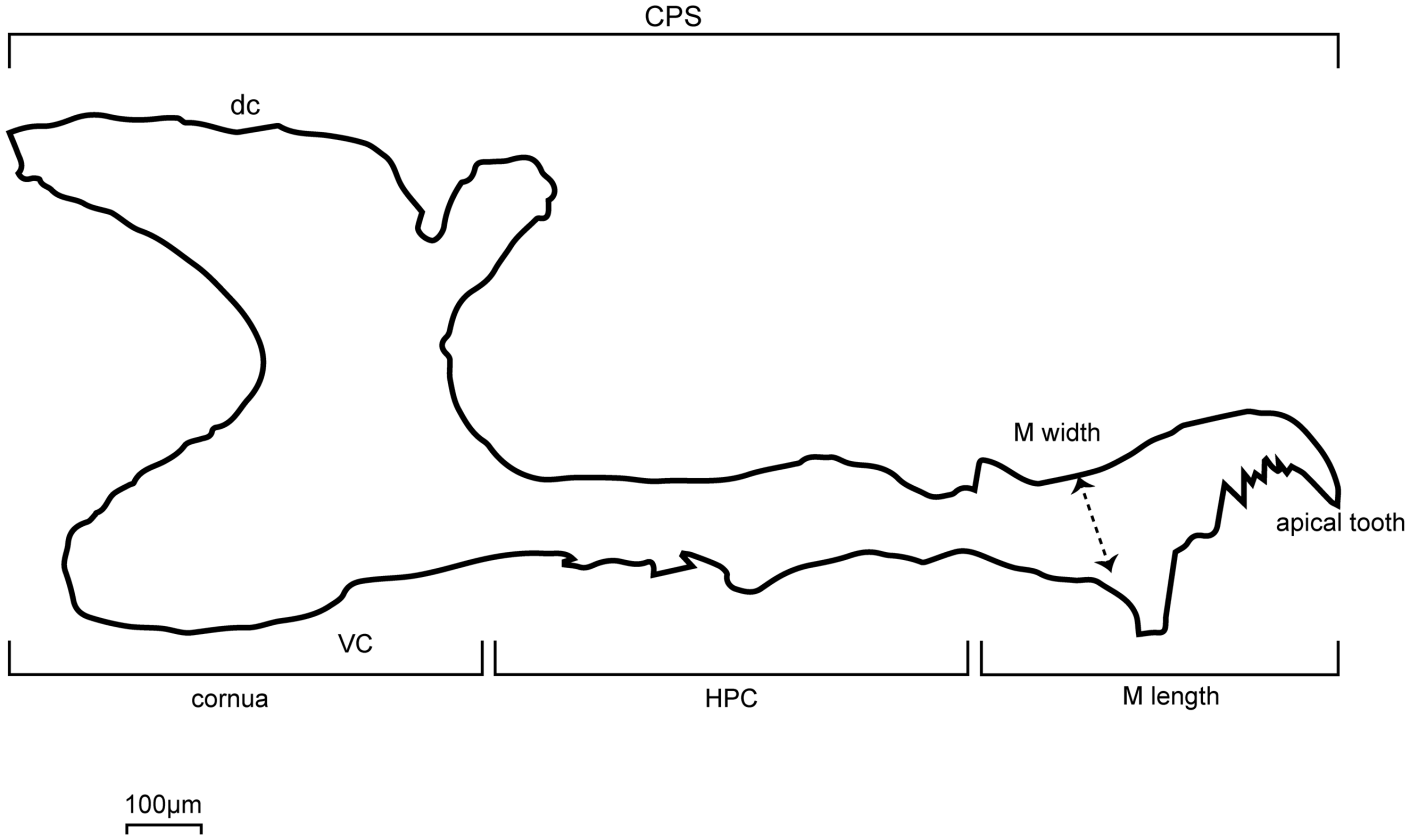
A simplified lateral view of Cephalopharyngeal skeleton CPS of *D*. *gaucha*. Hypopharyngeal sclerite and Conua HPC and Mandibles M are identified; dc, dorsal cornu; vc, ventral cornu. Measurement performed expressed in micrometers (μm) are framed. Larvae measured were 24, 48, 72, 96, 120, 144, 168 and 196 h old (N = 50 per larval age). HPC and M of larvae of the BA and CJ parental strains, reciprocal F1, two out of four F2 and four out of eight backcrosses were measured (N = 4,000 larvae). Mandible teeth number was also counted at each larval age. CPS shown corresponds to 96 h old larvae.

Interestingly, our findings indicate similar patterns of growth for the M and HPC (Figs 1A–1J to 3A–3J). These findings may be all signs of common genes participating in the development of these two very different anatomical parts (see Fig 4). Perhaps a single molecular function is common to the M and HPC development, as proposed by He and Zhang [28]. Pleiotropic genes tend to limit phenotypic variation, ensuring that the corporal organization is expressed in anatomic functioning wholes [5, 29]. Little attention has received the evolutionary role of pleiotropic genes in the development of organisms living in changing environments.

An appropriate integration of M and HPC guarantees the optimal performance of body parts necessaries to explore in a relatively short time ephemeral and variable environments as *Drosophila* breeding sites [16, 19]. *D*. *gaucha* larvae are highly mobile animals investing a substantial amount of time and energy searching for microorganisms to consume and places to pupate [19, 30]. Moreover, decaying cladodes of *Opuntia ficus-indica*, on which *D*. *gaucha* larvae breed in the nature, are filamentous. The fibers could offer resistance when they are ingested, requiring a fine integration of M and HPC, and pharynx to ensure that food reaches the larva gut. An epigenetically canalized development provides circumstances to assure a proper integration of those anatomic parts, guaranteeing food ingestion by the larvae.

Most studies on genetic recombination among isolates of one species have largely focused on consequences for phenotypic variation of such recombination [31, 32]. Our study suggests that inter-population recombination has also importance for the development and integration of body parts. In other investigations, we reported that hybridization of the BA and CJ strains affects the locomotion and feeding rate of *D*. *gaucha* larvae [30]. These behavioral changes could reflect alterations in M and HPC integration and/or neurological changes at central level. Consequently, recombination among isolates of one species may have consequences for the development of a diversity of traits ranging from behaviors to body parts that act cooperatively.

The epigenetics of post-embryonic development of morphological traits that do not play role in blocking gene flow, but are key to fitness have received little attention. The M and HPC are related to feeding, nutrition, and subsequently, fitness through a nexus of connections with rates of development, larval growth, adult body size, sexual success and fecundity. Our study suggests that the M and HPC represent an excellent model for any comprehension of the evolution of morphological organization.

### Concluding remarks

Our findings suggest integration of epigenetics networks in the post-embryonic development of body parts that act coordinately in *D*. *gaucha* larva. Such gene regulatory networks appear generate additivity, dominance, epistasis and pleiotropic phenomena. Notably, hybridization of two isolates of endemic *D*. *gaucha* seems to produce substantial changes in levels of gene expressions impacting the development of corporal structures. These epigenetic changes might be inherited by future generations providing a substrate upon which natural selection can act. Whereas we have not identified the molecules that regulate the post-embryonic development of M and HPC, we know that their interactions have ecological implications related with ingestion of food and presumably, in allocation of space and microhabitat selection as in pupation site preferences. Future molecular studies make feasible to better identify the epigenetic control of the development of M and HPC in *D*. *gaucha* larva. Those studies will also aid to better understand the nature of heritable epigenetic variation in natural populations of *D*. *gaucha* and their implications for morphological organization. In summary, our data suggest an inter-population evolutionary divergence in genetic regulation of development and functional integration of body parts essentials to ingest food and move. Such genetic differences between natural populations rarely are considered in evolution of the genus *Drosophila*.

## Materials and methods

### Subjects

*D*. *gaucha* is a South American neotropical species. Together with an additional 9 to 12 species, *D*. *gaucha* forms the *mesophragmatica* group of species of *Drosophila* [20, 21, 33]. We tested wild-type larvae of two natural *D*. *gaucha* populations (the Buenos Aires BA and Campos de Jordan CJ strains). Campos de Jordan, 22°44' S at 1700 meters sea level, has tropical height climatic conditions. The annual rainfall is approximately 1566 millimetres and the annual mean temperature is 13.6°C. Campos de Jordan is the only place in Brazil where snow occasionally falls in winter. Buenos Aires, 35°0' S at 25 meters sea level, has temperate humid climatic conditions. The annual rainfall is circa 1147 millimetres. Each strain was founded with about 25 flies (Campos de Jordan and Buenos Aires strains), and the sex ratio was variable. When the crosses were made, approximately 10 generations of breeding in the laboratory had elapsed. We presumed that a significant amount of the genetic variation present in the founders was retained when the strains were crossed. The F_**1**_, F_**2**_ and backcross adult individuals were all vigorous and female fecundity similar to that of the parental. Flies were all reared under constant light at 18 ± 1°C 80% humidity in 250 cc glass bottles in synthetic Burdick's medium [34]. *D*. *gaucha* grows better at this temperature and humidity than at 24°C. Facilities to change the light / dark cycle were not available in the laboratory. All experimental flies were raised and stored under the same conditions.

Groups of 20–30 inseminated females of the BA and CJ strains, and as well as the hybrids between the strains were allowed to lay eggs for 3–4 h on their respective plastic spoons filled with Burdick's medium. The spoons with eggs were incubated at 18°C. In *D*. *gaucha*, larvae rise from eggs out 48 h after they are deposited on the medium. We randomly collected larvae in 4-h windows every day for 8 days at 1–8 days after hatching. Thus, we examined the larvae of 1-day-old to 8-day-old larvae. We examined larvae of the two reciprocal F_**1**_, two out four reciprocal F_**2**_ and four out eight types of backcrosses, that is, 10 groups of genotypes.

### Hypopharyngeal sclerite and cornua and mandible measures

We measured M and HPC of *D*. *gaucha* larva [12, 35]. We measured length and width of M in micrometers (μm). Only the length (μm) of HPC was measured. HPC width changes notably at the two ends (Fig 4). This Fig 4 agrees almost exactly with Fig 1 published by Alvarez *et al*. [22]. However, Fig 1 in Alvarez *et al*. [22] contains some inaccuracies mainly in the names; these were corrected as shown in Fig 4 of the present manuscript. Table 3 describes the measurements performed on 24, 48, 72, 96, 120, 144, 168 and 192-hour-old larvae, covering the entirely larval period (N = 50 larvae per age and genotypic group). The goal was evaluate the effect of hybridization between the BA and CJ parental strains on the growth of those body parts.

The larvae were randomly collected and sacrificed by placing in phosphate-buffered saline PBS 0.1 M^-1^. Once dead, the larvae remained completely extended. The larvae were individually collected from the buffer and dried by depositing them on Whatman cellulose filter paper. Then, M and HPC were dissected and mounted as described by Frías *et al*. [35]. For this task we used a stereomicroscope Leica MZ6 at 20 x magnification.

### Crosses and statistical analysis

We crossed the CJ and BA strains reciprocally (see above). For the valid application of the Mather and Jinks model [23], the assumption of additivity must be tested. We attempted to remove multiplicative effects by changing the scale. We transformed the data to logarithms; this scale is used to convert multiplicative into additive effects [36]. However, log transformations did not remove multiplicative effects. We accepted that the collected data have nonlinear components. We followed the analysis, applying scaling tests to examine the adequacy of the results in an additive-dominance model [23]. The tests consider the relationships between the generation means. The failure to observe the relationships between the expected means for an additive- dominance model is indicative of non-allelic interactions [23]. We applied the individual scaling tests and the joint scaling test to ensure that the data contained non-allelic components [37].

We also used ANOVA to compare the parental and F_**1**_ generations. We tested whether the parental strains differed, whether there were reciprocal differences in the F_**1**_'s, and whether F_**1**_'s showed dominance. To determine whether there were maternal or sex—linkage effects we performed ANOVA for all 10 crosses (see Fig 1A–1J to 3A–3J). Thus we compare the CJ and BA parental, F_**1**_'s, F_**2**_'s and reciprocal backcrosses. For example, we tested whether larvae of backcross F_**1**_ males to CJ females had different larval mandible sizes compared with those from the reciprocal cross F_**1**_ females to CJ males at the same larval age.

We searched for the curve that mathematically best described changes in the traits measured (see above) as larval development proceeded. The function that showed higher *R*^*2*^ determination coefficient was chosen as the most reliable to describe the data (see S3 Table). We applied an ANOVA to compare the slopes of the traits measured in the parental strains and the F_**1**_, F_**2**_ and backcrosses.

We applied a multiple linear regression and **χ**^**2**^ test to estimate goodness-of-fit between an additive-dominant-epistatic model and the data (see S1 Table). Once applied the test, we estimated the [*m*], [*a*], [*d*], [*aa*], [*ad*] and [*dd*] parameters [23, 37].

To make our statistical analysis we used STATGRAPHICS Program, Centurion XVII 64-bit, paid 2014 version.

## Supporting information

S1 Fig Growth in width (μm) of mandibles M in BA, A; CJ, B; F_1_, C and D; F_2_, E and F, and backcross larvae, G—J of *D*. *gaucha*. For further details see Figs 1 and 2, and Table 2. See also reference [22].
(TIFF)

S1 Table Goodness of fit of an additive-dominant-epistatic model to the collected data. (DOCX)

S2 Table Statistical significance, *t*-test, of parameters value presented in Table 2 (see also Materials and methods). (DOCX)

S3 Table Proportion of variances, *R*^*2*^ –values, in the dependent variable that is predictable from the independent variable in the parental, F_1_, F_2_ and backcross generations. (DOCX)

S1 Data Set Data Godoy-Herrera et. al. MS. The genetics and development of mandibles M and hypopharyngeal sclerite and cornua HPC in larvae of *Drosophila gaucha*.
(XLS)
