# Supplementary figures and images for: The genetics and development of mandibles and hypopharyngeal sclerite and cornua in larvae of Drosophila gaucha
Source: PLoS One. 2017 Oct 18;12(10):e0185054. doi: 10.1371/journal.pone.0185054 (PMC5646785; doi:10.1371/journal.pone.0185054)

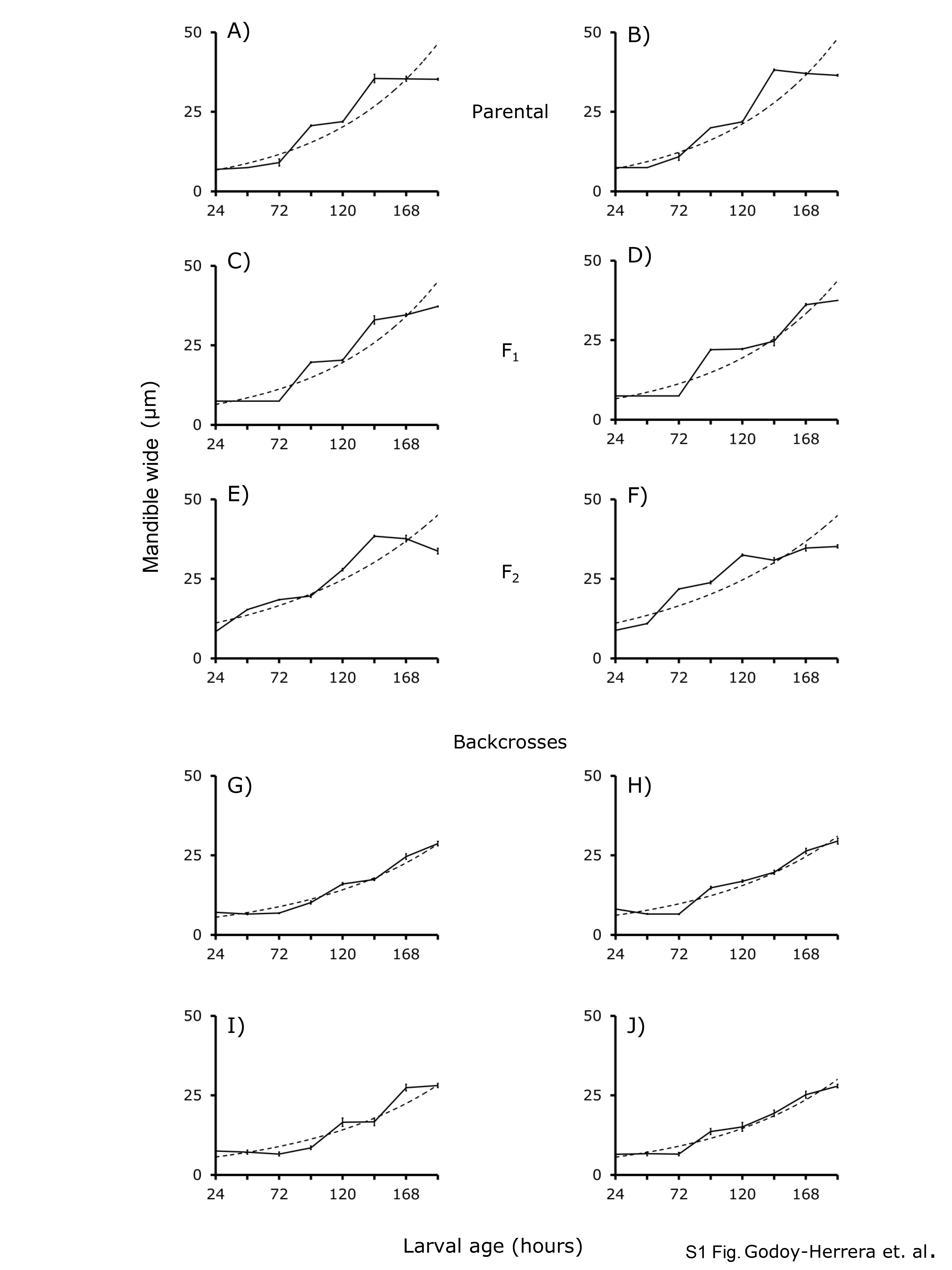

Supplement: S1 Fig — For further details see Figs 1 and 2, and Table 2. See also reference [22]. (TIFF) [file pone.0185054.s001.TIFF]
