# Supplementary material for: The genetics and development of mandibles and hypopharyngeal sclerite and cornua in larvae of Drosophila gaucha
Source: PLoS One. 2017 Oct 18;12(10):e0185054. doi: 10.1371/journal.pone.0185054 (PMC5646785; doi:10.1371/journal.pone.0185054)
Supplement: S1 Table — (DOCX) [file pone.0185054.s002.docx]

S1 Table. Goodness of fit of an additive- dominant- epistatic model to the collected data. A Chi-squared test was applied to evaluate significance of differences between the collected data and the model. For all crosses the parental lines were the Buenos Aires BA and Campos de Jordan CJ strains (see Materials and Methods).
