# Supplementary material for: The genetics and development of mandibles and hypopharyngeal sclerite and cornua in larvae of Drosophila gaucha
Source: PLoS One. 2017 Oct 18;12(10):e0185054. doi: 10.1371/journal.pone.0185054 (PMC5646785; doi:10.1371/journal.pone.0185054)
Supplement: S2 Table — (DOCX) [file pone.0185054.s003.docx]

S2 Table. Statistical significance, *t*-test, of parameter values presented in Table 3 (see also Materials and Methods).

[m] = Common effects to every genotype; [a] = Additive component; [d] = Dominant effects of means; [aa] = additive x additive interaction; [ad] = additive x dominance interaction; [dd] = dominance x dominance interaction.

*P < 0.01
