# Supplementary material for: The genetics and development of mandibles and hypopharyngeal sclerite and cornua in larvae of Drosophila gaucha
Source: PLoS One. 2017 Oct 18;12(10):e0185054. doi: 10.1371/journal.pone.0185054 (PMC5646785; doi:10.1371/journal.pone.0185054)
Supplement: S3 Table — (DOCX) [file pone.0185054.s004.docx]

| S3 Table. Proportion of variance, *R^2^*-values, in the dependent variable that is predictable from the independent variable in the parental, F_1_, F_2_ and backcross generations. The *R^2^* coefficient of determination shown correspond to length-wide growth of mandible of *D. gaucha* larvae; they were obtained from, respectively, the exponential and logarithmic curves. Comparable results were obtained for mandible width-wide growth and length-wide growth of Hypopharyngeal sclerite and cornua HPC (further details in Materials and Methods) . The *R^2^*-values for the reciprocal crosses are shown separately. BA, the Buenos aires parental strain; CJ, the Campos de Jordan parental strain. For all crosses, the first parent shown is the female. |
| --- |
|  |
|  |
|  |
